# Huanglian Jiedu Decoction ameliorates DSS-induced colitis in mice via the JAK2/STAT3 signalling pathway

**DOI:** 10.1186/s13020-020-00327-9

**Published:** 2020-05-08

**Authors:** Zhuo Lu, Wanna Xiong, Simeng Xiao, Yilong Lin, Kai Yu, Guihua Yue, Qiaoming Liu, Fang Li, Jianqin Liang

**Affiliations:** 1 Guangxi University of Chinese Medicine, Nanning, 530001 China; 2 Department of Pharmacy, Guangxi Medical College, Nanning, 530023 China; 3 College of Animal Science and Technology, Guangxi University, Nanning, 530004 China

**Keywords:** Ulcerative colitis, Huanglian Jiedu Decoction, JAK2/STAT3, Dextran sulphate sodium

## Abstract

**Background:**

Ulcerative colitis (UC) is an intestinal disease which was characterized by intestinal inflammation, mucosal injury and fibrosis. In this paper, the effect of Huanglian Jiedu Decoction (HJD), a well-known traditional Chinese medicine with significant anti-inflammatory effect, on dextran sulphate sodium (DSS)-induced UC in mice and inhibition of JAK2/STAT3 pathway were investigated.

**Methods:**

BALB/c mice were randomly divided into 6 groups: HJD group (high, medium and low dose), USAN group, UC group, and control group. UC in mice were induced through free access to 3% DSS solution. After being treated with HJD for 8 days, all animals were sacrifice. Pathological examination of colonic specimen was performed by H&E staining. Cytokines (TNF-α, IL-6, and IL-1β) in colon were assayed by ELISA and immunofluorescence, MPO in colon and ATT in serum were detected by ELISA. Moreover, mice in HJD group and UC group were treated with AG490 to inhibit the expression of JAK2 protein, then the expression of JAK2 and STAT3 protein in colon was determined by western blotting and immunofluorescence staining. Furthermore, KI67 in colon was examined by immunohistochemistry, and apoptosis was detected by TUNEL staining, and collagen deposition was assayed by Masson staining after JAK2/STAT3 pathway in UC mice was inhibited by HJD.

**Results:**

After mice being treated with HJD, the symptoms (weight loss and haematochezia) of UC were alleviated, and the contents of inflammatory cytokines (TNF-α, IL-6 and IL-1β) and MPO in colon were significantly decreased. The expression of JAK2 and STAT3 protein was reduced after administration with HJD. After JAK2/STAT3 pathway being inhibited with HJD, the cell apoptosis, collagen deposition and immunoreactivity of macrophage in colon were significantly reduced, but the expression of Ki67 was markedly enhanced in both UC group and HJD group compare with control group.

**Conclusions:**

HJD treatment can alleviate intestinal mucosal damage and has the protective effect on UC by downregulating JAK2 and STAT3 expression to reduce inflammation via JAK2/STAT3 pathway.

## Background

Ulcerative colitis (UC) is an intestinal disease that is characterized by intestinal inflammation, mucosal injury, and fibrosis. It will be prone to develop to colorectal cancer with a probability about 5%–10% in the development of “inflammation-atypical hyperplasia-cancer” [1, 2]. 5-ASA, immunosuppressive agents, monoclonal antibodies (Infliximab), and glucocorticoids are the effective therapeutic drugs for UC. However, after a long-term use of these drugs, it is easy to bring toxicity, allergy, upper gastrointestinal bleeding, and other side effects [3].

The etiology and pathogenesis of UC remains unknown. It is commonly believed that the abnormalities of intestinal immunity and intestinal flora balance in the early stage of UC were the pathogenesis [4, 5]. Intestinal mucosal damage usually leads to a burst of cytokines (IL-6, IL-1β, TNF-α, etc.). The excessive expression of IL-6 can deepen the infiltration of macrophages and neutrophils, and also promote cell apoptosis and autophagy via JAK2/STAT3 pathway, which results in much severer damage [6]. The intestinal impairment was associated with the abnormally expression of STAT3 and JAK2 protein [7, 8].

HJD consists of four herbal materials, including *Rhizoma coptidis* (HL), *Cortex phellodendri chinensis* (HB), *Radix scutellariae* (HQ) and *Fructus gardeniae* (ZZ)), which are widely used in the treatment of sepsis, arthritis, autoimmune diseases, and intestinal diseases in clinical practice [9–11]. Its anti-inflammatory effects could be related to the action on multiple protein targets [12]. However, the therapeutic effect of HJD on UC and its mechanism are still unclear. In this paper, the effect of HJD on UC and its regulation on the JAK2/STAT3 pathway were investigated, the effects of HJD on the apoptosis and proliferation which were regulated by JAK2/STAT3 pathway in colon were detected.

## Methods

### Chemical reagents

Dextran sulphate sodium (DSS; molecular weight 36,000–50,000 Da, HPLC ≥ 97% purity) was purchased from MP Biomedicals Inc. (Santa Ana, CA, USA). Dimethyl sulphoxide (DMSO) and Tyrphostin AG490 were purchased from Sigma-Aldrich (St. Louis, Missouri, USA). OB reagent was purchased from Zhuhai Besso Biotechnology Company (Zhuhai, China). Mesalazine (USAN; L/N: 16J05289L) was purchased from Losan Pharma GmbH (Frankfurt, Germany).

### Preparation of HJD

HL, HB, HQ and ZZ were purchased from Nanning Wanyaotang Pharmaceutical *Co., Ltd.* (Nanning, China) and authenticated by Professor Songji Wei (College of Pharmacy, Guangxi University of Chinese Medicine, China). The voucher specimens, deposited at the Guangxi University of Chinese Medicine, were HL-2017-0401, HQ-2017-0402, HB-2017-0403, and ZZ-2017-0104 for HL, HQ, HB, and ZZ, respectively.

Briefly, 300 g of HL, 200 g of HB, 200 g of HQ, and 300 g of ZZ were extracted twice, for 2 h each time, by refluxing in water according to the weight ratio of 15:1 of water to herbal. Then, the aqueous solution was combined, then filtered and concentrated in a rotary evaporator to a fluid extract with a relative density of about 1.05–1.20 (measured at 50–60 ℃), and then was stored in a refrigerator at 4 ℃. The content of berberine hydrochloride, a kind of active compound, in HJD extract was 20.39 mg/g, which was analyzed by high-performance liquid chromatography (Alliance 2695, Waters, USA) on a Inertsil ODS-2 C18 analytical column (4.6 mm × 250 mm, 5 μm) with elution by acetonitrile-0.05 moL/L phosphoric acid aqueous solution (50:50), a flow rate at 1.0 mL/min and the detection wavelength of 345 nm. The column temperature was 30 ℃, and the injection volume was 10 μL.

### Animal treatment

Four-week-old male BALB/c mice (18 ± 2 g) were purchased from Hunan SJA Laboratory Animal *Co., Ltd.* (Hunan, China) with license No. SCXK (Hunan) 2016–0002. All mice were fed at 25 ℃ ± 2 ℃ and 50% ± 5% relative humidity (RH) with a 12-h light/dark cycle with free access to standard water and food.

In the pharmacodynamics experiment, mice (n = 60) were randomly divided into 6 groups, namely the control group (Control; 10 mL/kg, 0.9% saline), UC group (UC; 3% DSS), high-HJD dose group (H-HJD; 24.66 g/kg, weight ratio between crude drug and mice), medium-HJD dose group (M-HJD; 12.33 g/kg), low-HJD dose group (L-HJD; 6.17 g/kg), and USAN group (USAN; 0.3 g/kg). In the JAK2/STAT3 pathway blocking experiments, mice (n = 60) were randomly divided into a control group (Control; 10 mL/kg, 0.9% saline), UC group (UC; 3% DSS), AG490 group (AG490; 0.01 g/kg), HJD group (HJD; 24.66 g/kg), and interference group (AG490 + HJD; 0.01 g/kg AG490 + 24.66 g/kg HJD). Each group consisted of 10 mice.

After 3 days of acclimation, mice free drank 3% DSS solution (w/w) to induce UC except the mice in control group. For the HJD treatment group and USAN group, HJD and USAN were administrated by gavage once a day at a volume of 10 mL/kg. The AG490 group was subjected to intraperitoneal injection. The UC group and control group were subjected to gastric lavage with normal saline. All mice were treated for 8 days.

Then, mice were euthanized by cervical dislocation on day 8. Serum was obtained after centrifugation at 3000 rpm for 10 min at 4 ℃ and was stored at − 20 ℃. Colon tissues from the anus to the ileum were collected and cut in half. One half was fixed in 4% neutral-buffered formalin for histopathological and immunohistochemistry (IHC) analysis, the other half was frozen in liquid nitrogen and stored at − 80 ℃ for western blotting assays.

### Colon macroscopic damage index (CMDI)

The congestion, oedema, atrophy, and ulcer of the colon were surveyed after mice had been dissected, and CMDI was evaluated according to the previously published criteria [13]: 0: no obvious congestion, oedema or ulcer; 1: local congestion, oedema but no ulcer; 2: scattered ulcer spots; 3: linear continuous distribution of ulcer points, inflammatory oedema, hyperaemia < 1 cm; 4: formation of ulcer lesions, inflammatory oedema, hyperaemia range > 1 cm.

### Disease activity index (DAI)

All mice were weighed, the faecal characteristics were observed, and faecal occult blood tests were performed every day. The DAI was evaluated according to a previously published criteria [14]: 0: 0% weight loss, normal stool and negative occult blood test; 1: 1–5% weight loss, a normal stool, and negative occult blood test; 2: 6–9% weight loss, a soft stool, and positive occult blood test; 3: 10–15% weight loss, a soft stool, and positive occult blood test; 4: > 15% weight loss, diarrhoea, and visible bleeding.

### Haematoxylin and eosin (H&E) staining

Colon tissues were fixed with 4% neutral-buffered formalin for 24 h. After that the colon was eluted with gradient ethanol and then immersed in xylene for 1 h. The paraffin-embedded colon samples were sliced into 4-μm-thick sections, stained with haematoxylin and eosin, imaged under a light microscope for routine histopathological examination, and then graded according to the scoring criteria [15]: 0: no inflammatory cell infiltration, no goblet cell damage; 1: inflammatory cell immersion and local loss of goblet cells in the mucosal layer; 2: inflammatory infiltration in the mucosa, spreading to the submucosa, and loss of multiple sites of goblet cells; 3: inflammation continuously distributed in the mucosa and submucosa, with the loss of a large number of goblet cells; 4: full-thickness inflammatory cells seriously infiltrated and goblet cells completely damaged.

### Enzyme-linked immunosorbent assay (ELISA)

The contents of TNF-α, IL-6, IL-1β and MPO in the colonic tissue, and α-1 antitrypsin (ATT) in serum (Nanjing Jiancheng Bioengineering Institute, China) were determined by ELISA according to the manufacturer’s instructions.

### Western blotting (WB)

The colon tissue was added to radioimmunoprecipitation assay lysate (containing a protease inhibitor), and the supernatant was drained after homogenization. The protein concentration was determined using the bicinchoninic acid method after gradient dilution. The proteins were separated by SDS-PAGE and transferred to a polyvinylidene difluoride membrane (Millipore, USA). Membranes was sealed at room temperature for 1 h and then incubated with primary antibodies against JAK2 (D2E12, Cell Signaling Technology, CST Inc., Beverly, MA, USA), STAT3 (124H6, CST Inc.), and phospho-STAT3 (Ser727, CST Inc.) overnight at 4 ℃. Then the membranes were washed 3 times and incubated with a horseradish peroxidase (HRP)-conjugated secondary antibody for 1 h at room temperature (RT). The bands were semi-quantitated with Image J (National Institutes of Health, USA) software.

### Immunohistochemistry (IHC)

The 4-μm-thick slices were mounted on glass slides and baked for 1.5 h at 50 ℃, after which they were deparaffinized and the endogenous peroxidase activity quenched. The primary antibody Ki67 (ZA-502, 1:1000 dilution) was incubated on the slides for 12 h at 4 ℃. After rinsing three times with phosphate-buffered saline solution containing Tween, the horseradish peroxidase-conjugated secondary antibody (ZSGB-BIO, PV-9001) was incubated for 20 min at RT and then visualized after incubation with 3, 3-diaminobenzidine for 10 min at RT, after which the binding sites were sealed with neutral resin. Terminal deoxynucleotidyl transferase dUTP nick end labelling (TUNEL) analysis was performed on the colon tissue using an In-Situ Cell Death Detection Kit (Roche, 1:100 dilution). Images were obtained at 20× the original magnification. The average optical density (AOD) was measured by Image J software.

### Immunofluorescence staining

Colon tissue was sealed with wax and sectioned into 4 μm thick slices. Sectioned samples were deparaffinized in xylene, rehydrated with gradient alcohol, and subjected to antigen retrieval. Colon sections were washed with PBS five times for 3 min each. Then, 0.5% (wt/vol) Triton X-100 and blocking serum were added successively and incubated for 10 min and 1.5 h, respectively. The tissue was incubated in primary antibody, rabbit TNF-α (1:200, AF7014, Affinity Biosciences Inc., China), rabbit IL1-β(1:200, AF5103, Affinity Biosciences Inc., China), rabbit IL-6 (1:200, DF6087, Affinity Biosciences Inc., China), rabbit JAK2 (1:200, AF6022, Affinity Biosciences Inc., China), rabbit STAT3 (1:200, AF6294, Affinity Biosciences Inc., China), rabbit F4/80(1:200, DF7762, Affinity Biosciences Inc., China), at 4 ℃ overnight. After being washed four times with PBS, the sections were incubated with the secondary antibody (ZF-0516, Alexa Fluor^®^ 594, ZSGB-Bio Inc., China) for 2 h at RT and protected from light. Images were obtained at 20× the original magnification. The relative area immunoreactivity was calculated with Image J software.

### Masson staining

After dewaxing, sectioned samples was stained with hematoxylin for 30 s, hydrochloric acid to induce differentiation into blue, Masson compound staining solution for 2 min, 1% phosphomolybdate and bright green staining solution for 5 min, respectively. The sections were washed 3 times with 0.2% acetic acid after each staining, then dehydrated with anhydrous alcohol, cleaned with xylene until transparent, and sealed tightly with neutral glue. Images were obtained at 20× and 40× the original magnification. The collagen fibbers in tunica mucosa and submucosa was calculated with Image J software.

### Statistical analysis

Statistical data are expressed as mean ± standard deviation (SD), and a one-way analysis of variance (ANOVA) followed by Dunnett’s t-test was used for comparisons between groups. The data were analysed with trial version SPSS (SPSS Inc., Chicago, IL, USA). *P* < 0.05 was regarded as statistically significant.

## Results

### HJD treatment attenuated the symptom in DSS-induced UC mice

**Fig. 1:**
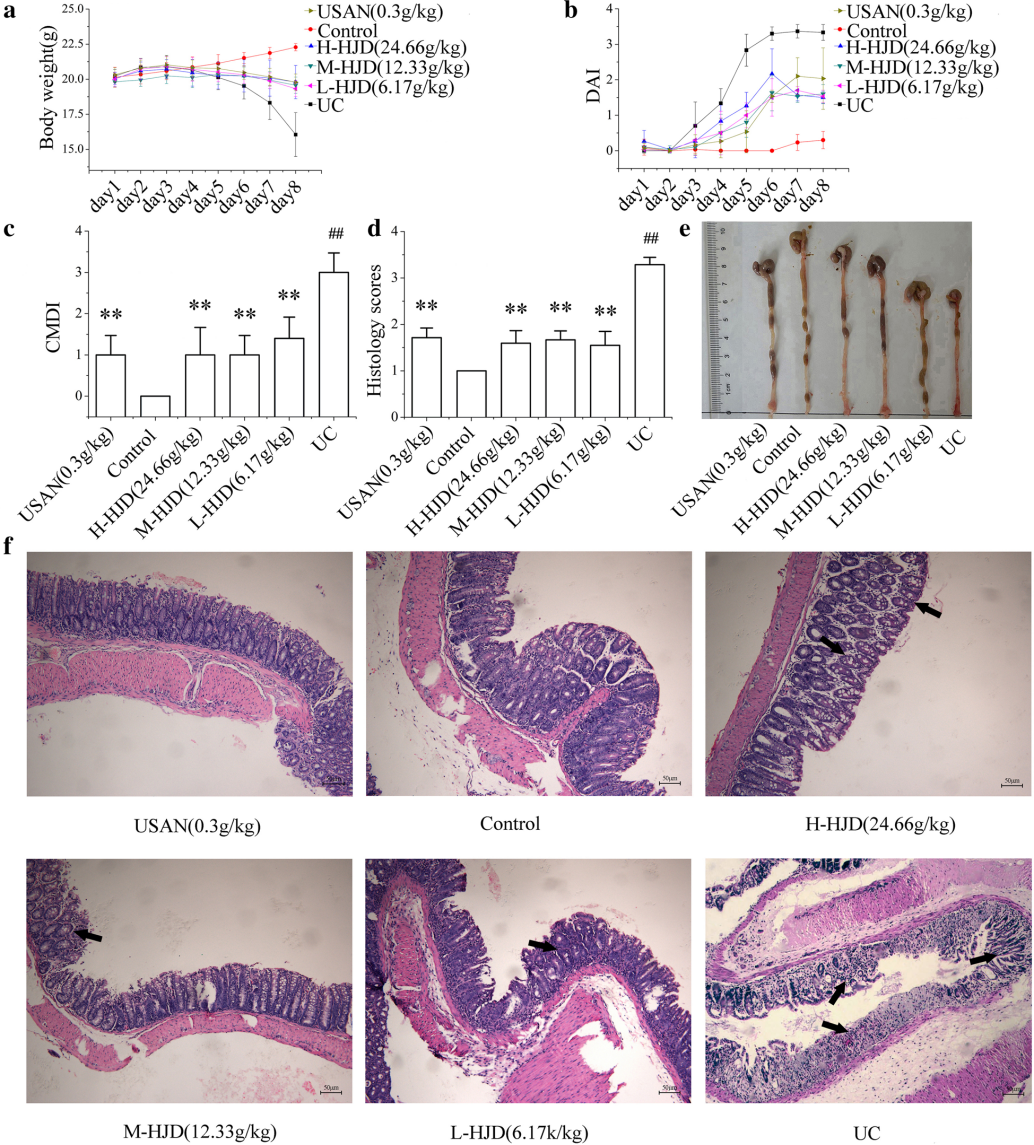
Protective effect of HJD against DSS-induced UC in mice. **a** Body weight. **b** DAI. **c** CMDI. **d** Histology score of the colon calculated by H&E staining. **e** Colon macroscopic damage and length. **f** Image of H&E staining obtained at 20× magnification in the colon. All of the data are expressed as mean ± SD (n = 10), **P < 0.01 compared with UC group, and ^##^*P* < 0.01 compared with control group. Scale bar = 50 μm

Contrary to the stable increase in body weight, soft stool, and negative occult blood test in control group, there were significant changes in UC group on the 5th day. The weight loss of the mice in HJD group and USAN group was less significant than in UC group (Fig. 1a). Diarrhoea and haematochezia were found in the groups treated with 3% DSS (UC group, HJD group and USAN group) on days 3 to 8. The different was that diarrhoea and haematochezia in HJD group and USAN group was less severe than that in UC group. Therefore, the DAI was significantly increased in UC group compared with control group. Meanwhile, a lower DAI was demonstrated in HJD group and USAN group (Fig. 1b). Moreover, the CMDI and histology scores in UC group were significantly higher than that in other groups (Fig. 1c, d).

As shown in Fig. 1e, there was significant shortening of the colon length in UC group, with obvious congestion, oedema, and atrophy compared with control group. These symptoms were much less severe in HJD group and USAN group. These results indicated that HJD significantly ameliorated the symptoms of UC in mice, including weight loss, diarrhoea, haematochezia, and oedema. As shown by H&E staining (Fig. 1f), it exhibited an intact epithelial layer and crypt-villus structures in control group. However, the other groups exhibited histopathological injuries, including inflammatory cell infiltration and a paucity of intact crypt-villus structures. Interestingly, HJD and USAN significantly alleviated the intestinal damage through enhancing the intestinal mucosa integrity, and attenuating the inflammatory infiltration.

### HJD treatment reduced the production of cytokines (TNF-α, IL-6, and IL-1β) and MPO in colon tissues, and enhanced ATT level in serum

**Fig. 2:**
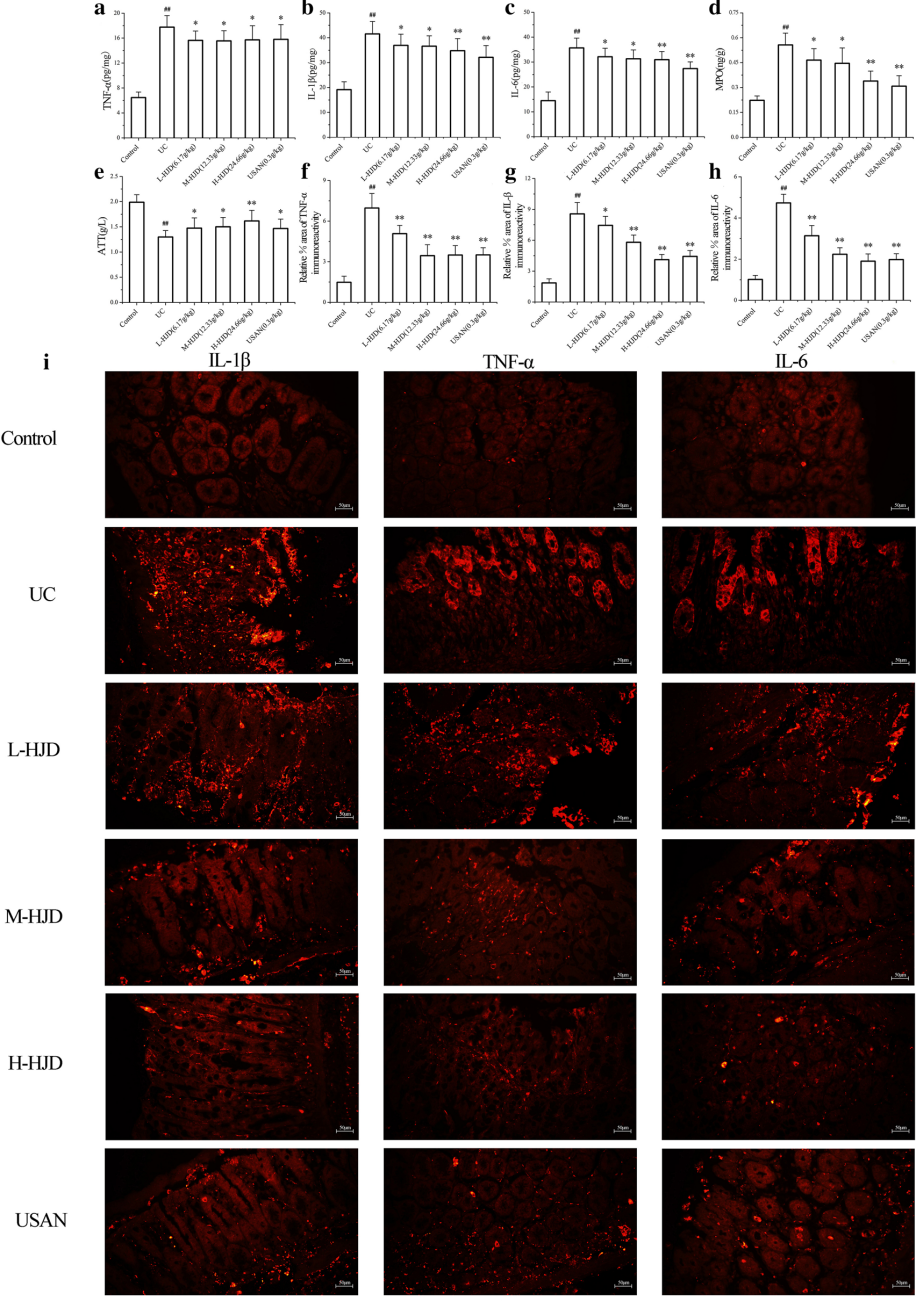
HJD downregulated the contents of TNF-α, IL-6, and IL-1β in colons and increased ATT content in serum. **a**–**e** Contents of TNF-α, IL-1β, IL-6, MPO and ATT. **f**–**i** Immunofluorescence staining of TNF-α, IL-1β and IL-6 in colon. *P < 0.05, **P < 0.01 compared with UC group, and ^##^*P* < 0.01 compared with control group. Scale bar = 50 μm

The results (Fig. 2a–c) showed that the contents of TNF-α, IL-6, and IL-1β in UC group were significantly increased compared with control group (*P* < 0.01). After mice being treated with USAN and HJD, the concentrations of TNF-α, IL-6, and IL-1β were significantly decreased compared with UC group (*P* < 0.05). In addition, we analyzed these proteins by immunofluorescence analysis, similar results were obtained (Fig. 2f–i). Moreover, as shown in Fig. 2d–e, HJD treatment MPO were significantly decreased increased the ATT level (*P* < 0.01).

### HJD inhibited the protein expression of JAK2 and STAT3

**Fig. 3:**
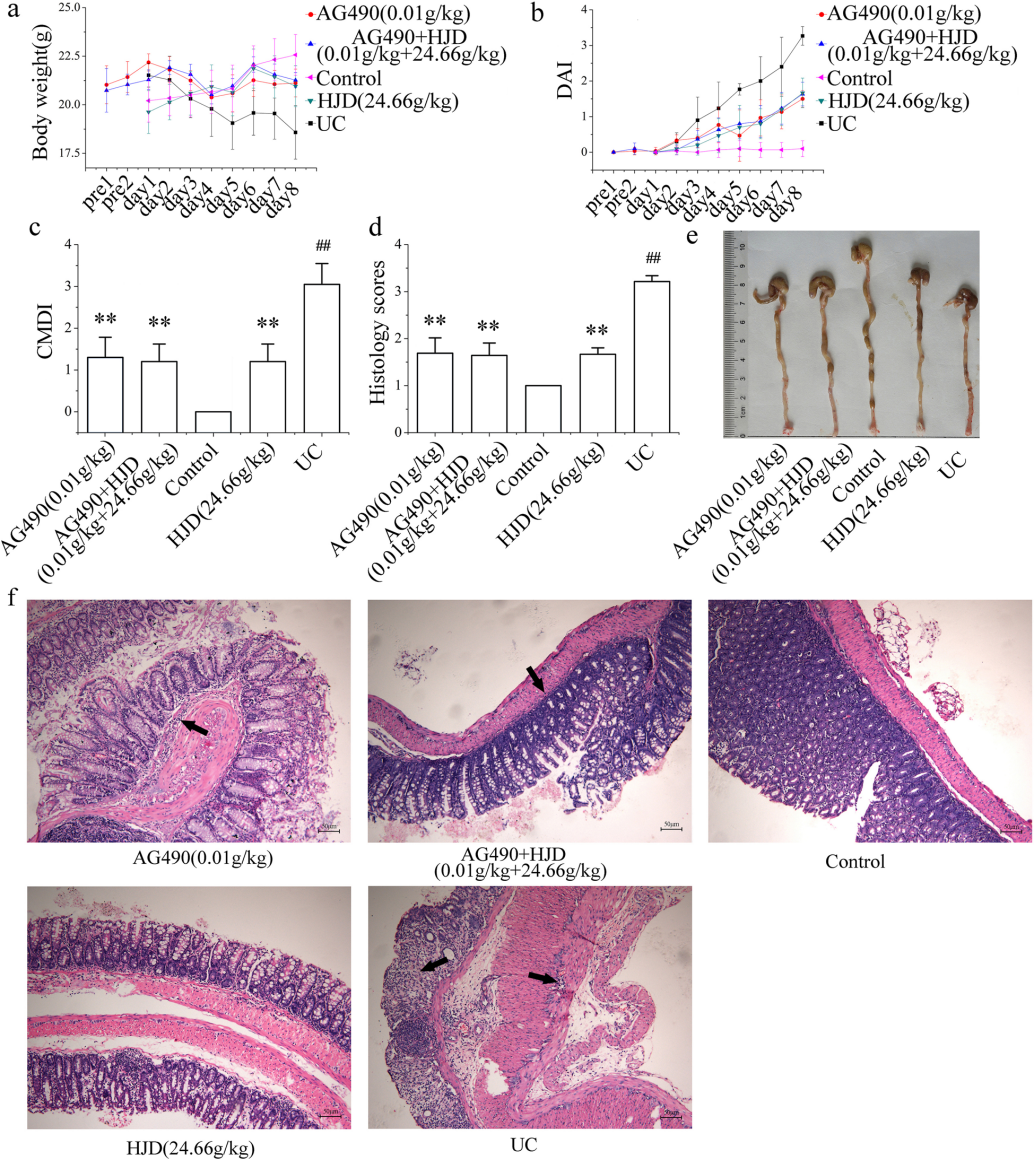
The effects of HJD and AG490 on UC in mice. **a** Body weight. **b** DAI. **c** CMDI. **d** Histology score of the colon calculated by H&E staining. **e** Macroscopic damage and length of colon. **f** Image of H&E staining obtained at 20× magnification. All of the data are expressed as mean ± SD (n = 10), **P < 0.01 compared with UC group; ^##^*P* < 0.01 compared with control group. Scale bar = 50 μm

AG490, a type of JAK2 inhibitor, was used to block the JAK2/STAT3 pathway. As shown in Fig. 3, the mice treated with AG490 (AG490 group and AG490 + HJD group), and HJD group significantly attenuated weight loss, diarrhoea, haematochezia, and oedema, with a lower CMDI and histology scores compared with UC group (*P* < 0.05). They exhibited a therapeutic effect to UC in mice.

**Fig. 4:**
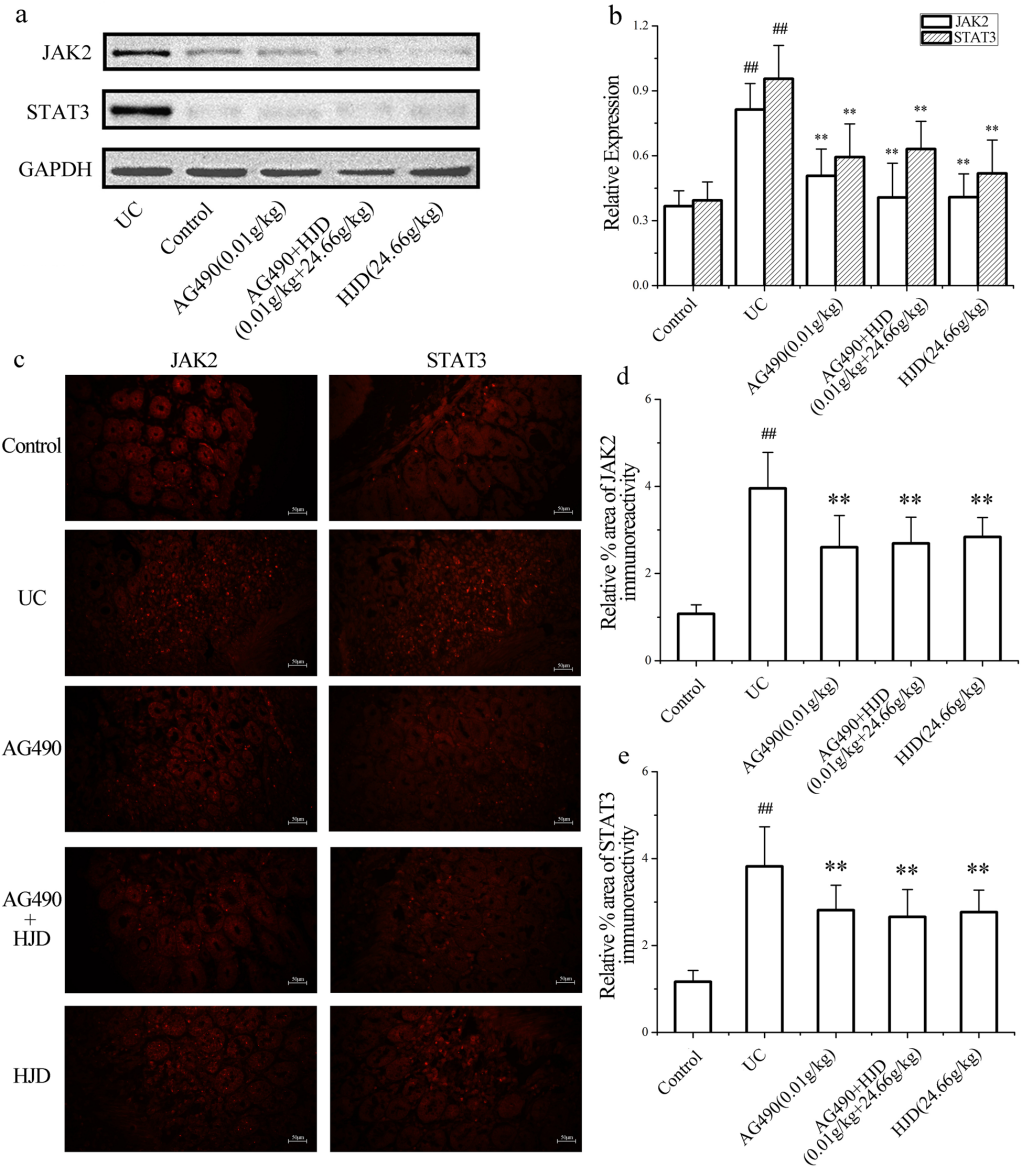
Effect of HJD on the protein expression of JAK2 and STAT3 in colon. **a** Western blotting of JAK2 and STAT3 protein. **b** Semi-quantitative analysis of JAK2 and STAT3 proteins. **c**, **d** Immunofluorescence staining of JAK2 and STAT3 co-localized in control group, UC group, AG490 group, AG490 + HJD group and HJD group. All data are expressed as mean ± SD, **P < 0.01 compared with UC group, and ^##^*P* < 0.01 compared with control group. Scale bar = 50 μm

WB assay (Fig. 4a, b) showed that the expression of JAK2 and STAT3 in UC group were significantly increased compared with control group (*P* < 0.01), while their expression in AG490 group and HJD group were significantly downregulated (*P* < 0.01). Similar results obtained by immunofluorescence analysis of JAK2 and STAT3 protein (Fig. 4c–e). This indicated that HJD exhibited an inhibition of JAK2/STAT3 pathway on UC in mice.

### Changes of KI67, apoptosis, collagen deposition and immunoreactivity of macrophage in colon after inhibition of JAK2/STAT3 pathway with HJD

**Fig. 5:**
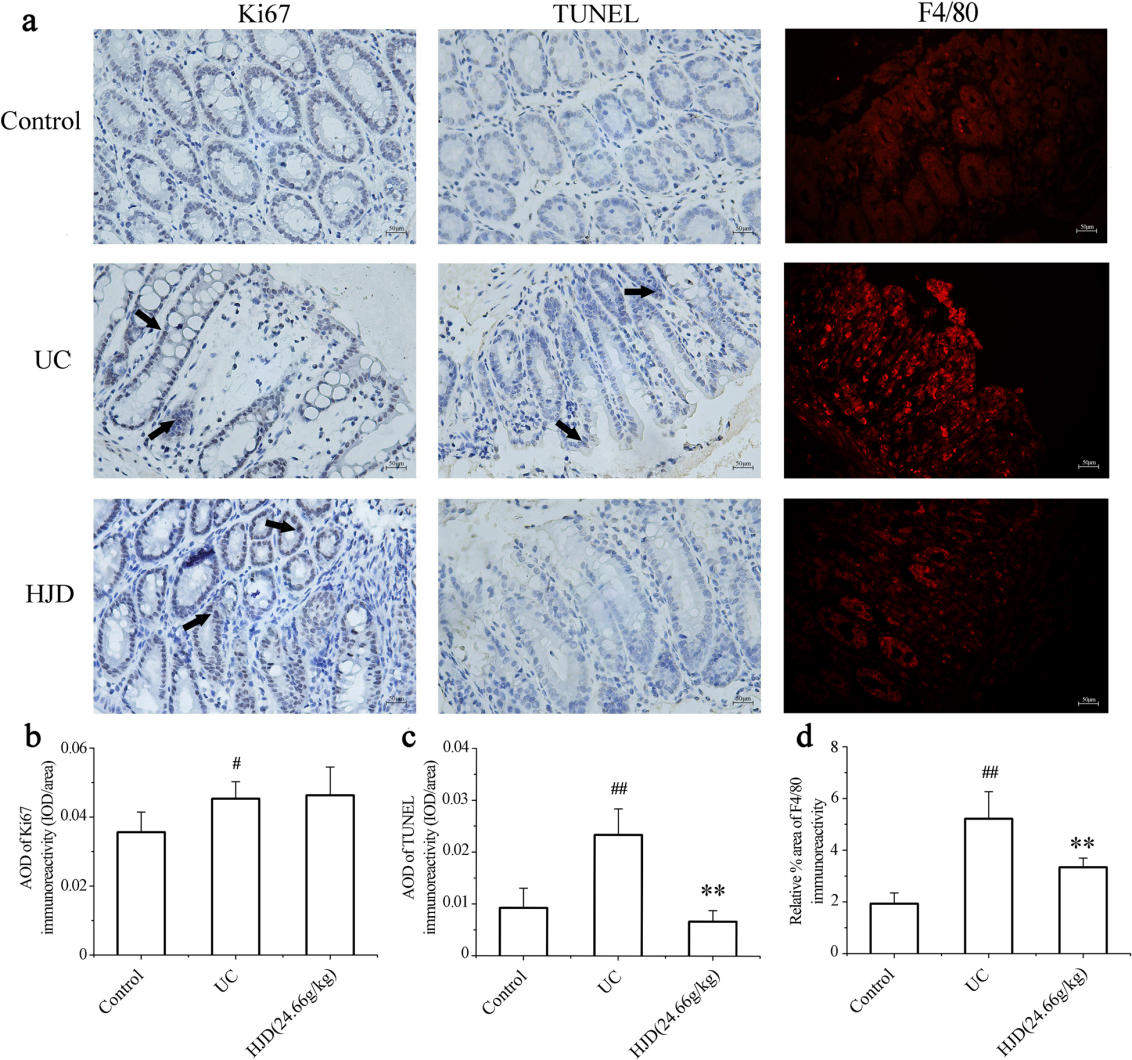
Effects on apoptosis, Ki67 expression and macrophages in colonic mucosa after HJD pre-treatment. **a** F4/80 by immunofluorescence, Ki67 expression by IHC staining, and apoptosis by TUNEL staining. **b** AOD of Ki67. **c** AOD of apoptosis. All of the data were expressed as mean ± SD. **d** Semi-quantitative analysis of immunofluorescence staining for F4/80 co-localized in UC group, control group and HJD group. All images were obtained at a magnification of 20× . **P < 0.01 compared with UC group, and ^#^*P* < 0.05, ^##^*P* < 0.01 compared with control group. Scale bar = 50 μm

Normal physiological proliferation, apoptosis, differentiation, migration, and tight junctions of intestinal epithelial cells, which are regulated through the JAK2/STAT3 pathway, are essential for maintaining the integrity of the intestinal mucosa. In this paper, IHC and TUNEL were applied to detect Ki67 expression and apoptosis in colonic epithelial cells, respectively. The results (Fig. 5a–c) showed an increase in Ki67 and a decrease in apoptosis in both UC group and HJD group compared with control group. To characterize the infiltrated macrophage population in the colon, immunofluorescence staining was performed for macrophages phenotypic markers F4/80. The results showed an increase of F4/80 macrophages in UC group compared with control group (*P* < 0.01), and a decrease of F4/80 macrophages was observed in HJD group (Fig. 5a, d).

**Fig. 6:**
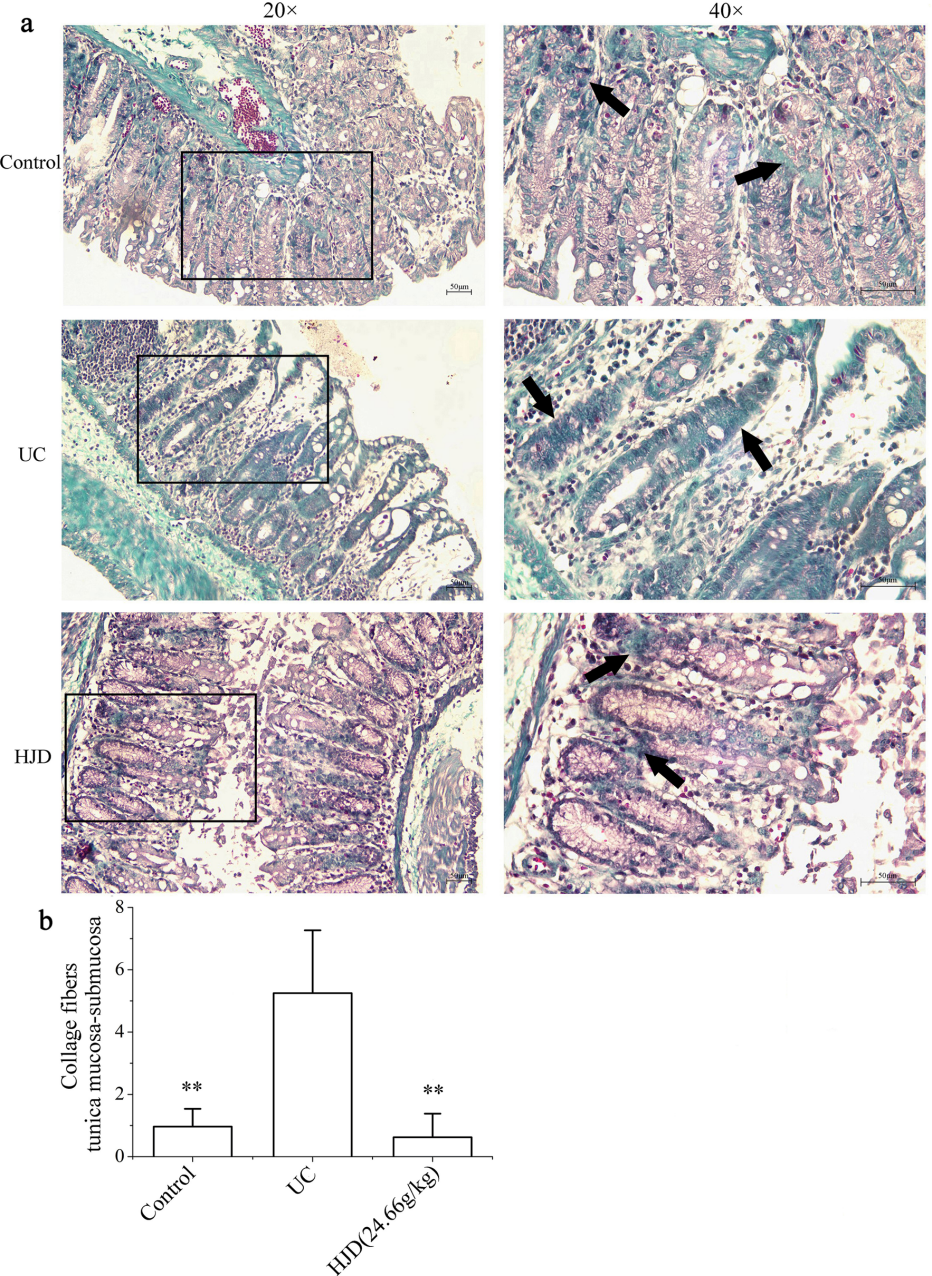
Effects on collagen expression in colonic mucosa of UC in mice after HJD pre-treatment. **a** Collagen deposition by Masson staining (blue). **b** IOD analysis. All of the data are expressed as mean ± SD. Images were obtained at a magnification of 20× or 40× . **P < 0.01 compared with UC group. Scale bar = 50 μm

Collagen fibrosis is a pathological manifestation of many diseases. The abnormal accumulation and degradation of collagen are the basis of fibrosis. It would bring about intestinal fibrosis in UC patient. Since apoptosis easily leads to the disruption of intestinal mucosal integrity and barrier function, which leads to inflammation and intestinal fibrosis, the expression of collagen in colonic mucosa of UC mice was performed by Masson staining. As shown in Fig. 6, the expression of collagen proteins in UC group was significantly increased (*P* < 0.01) compared with control group, while in HJD group it was significantly decreased (*P* < 0.01) compared with UC group. These results suggested that HJD treatment could reduce the risk of intestinal fibrosis in UC.

## Discussion

UC is characterized by intestinal inflammation and mucosal damage. The damage of intestinal mucosa is crucial to the occurrence and development of UC. The DSS-induced colitis mouse model was widely used to study the pathogenesis of UC and screen therapeutic drugs [16]. In the acute stage of UC, the release of inflammatory factors (TNF-α, IL-1β, and IL-6) will aggravate the accumulation and infiltration of inflammatory cells in colonic tissue [17, 18]. The excessive inflammation will further destroy the integrity of the intestinal barrier, which would lead to the formation of intestinal oedema and ulceration [7]. Inhibiting the expression of inflammatory cytokines will exhibit a therapeutic effect on UC [19–21]. Our results demonstrated that HJD treatment markedly alleviated colonic mucosal injury in UC mice, reduced the level of inflammatory cytokines (IL-1β, IL-6, and TNF-a) and MPO in colon, and increased the content of ATT.

JAK2/STAT3 pathway is an important and classical inflammatory pathway. JAK2 and STAT3 proteins are the key regulators of inducible expression of many genes that are involved in inflammatory responses in the intestinal tract [22, 23]. Overactivation of the JAK2/STAT3 pathway may have a negative effect on UC. In UC patients, the severity of the intestinal lesion was positively correlated with the expression of STAT3 [22]. To further elucidate the effect of HJD on JAK2/STAT3 pathway, AG490, a specific JAK2 inhibitor, was used to block the JAK2/STAT3 pathway [24]. We found that, similar to that reported about the overexpression of STAT3 and NF-KB caused abnormal activation of JAK2/STAT3 pathway [25], the expressions of JAK2 and STAT3 in the UC group were significantly increased. After administration with HJD, the expressions of JAK2 and STAT3 decreased significantly (*P* < 0.01). This suggested that HJD might inhibit JAK2/STAT3 pathway.

JAK/STAT pathway plays an important role in regulating apoptosis and proliferation. Thus, mono-antibodies of anti-TNF, such as adamumab (HUMIRA^®^) and infliximab (REMICADE^®^), are used for treatment of inflammatory bowel disease (IBD). Study showed that mono-antibodies inhibited apoptosis of intestinal epithelial cells and promoted mucosal repair [26]. Ki67, a marker of cell proliferation, is abnormally expressed in intestinal inflammation and carcinogenesis, and is associated with intestinal inflammation or proliferation, infiltration, and metastasis of malignant cells [27, 28]. In this paper, we found that Ki67 expression in UC group was significantly higher than control group (*P* < 0.05). However, an increase of Ki67 expression after administration of HJD was observed too. Furthermore, TUNEL staining was used to detect apoptosis of colon epithelial cells. Interestingly, the result showed that the apoptosis in UC group was significantly higher than that in control group (*P* < 0.01), meanwhile the apoptosis was significantly decreased after HJD intervention (*P* < 0.01). These results suggested that HJD could regulate cell proliferation and apoptosis through JAK2/STAT3 pathway [29].

In the development of IBD complicated with intestinal fibrosis, some fibrosis factors, such as transforming growth factor beta (TGF-β), would stimulate the disordered expression of extracellular matrix in the intestinal wall, and then cause intestinal stenosis and fibrosis [30]. This process is closely related to the expression of collagen in the intestinal mucosa. To investigate collagen deposition in the colonic mucosa in UC mice, Masson staining was used to detect collagen expression. The results showed that the collagen deposition in UC group was significantly higher than that in control group, which might be related to the long-term irritation caused by acute inflammation and cell proliferation in the intestinal mucosal. However, the deposition of collagen decreased significantly in HJD group (*P* < 0.01). These results suggest that HJD is beneficial for alleviating intestinal fibrosis. More experiments are necessary to study the mechanisms of HJD on cell proliferation and apoptosis.

## Conclusions

HJD treatment can alleviate intestinal mucosal damage and has the protective effect on UC by downregulating JAK2 and STAT3 expression to reduce inflammation via JAK2/STAT3 pathway.

## Data Availability

The datasets used in the current study are available from the corresponding author on reasonable request.

## Abbreviations

HJD: Huanglian Jiedu Decoction
HL: Rhizoma coptidis
HB: Cortex phellodendri chinensis
HQ: Radix scutellariae
ZZ: Fructus gardeniae
UC: Ulcerative colitis
DSS: Dextran sulphate sodium
USAN: Mesalazine
DAI: Disease activity index
CMDI: Colon macroscopic damage index
H&E: Haematoxylin and eosin
ELISA: Enzyme-linked immunosorbent assay
WB: Western blotting
TUNEL: Terminal deoxynucleotidyl transferase dUTP nick end labelling
5-ASA: 5-aminosalicylic acid
IBD: Inflammatory bowel disease
CMDI: Colon macroscopic damage index
IHC: Immunohistochemistry
RIPA: Radioimmunoprecipitation assay
PVDF: Polyvinylidene difluoride
HRP: Horseradish peroxidase
RT: Room temperature
RH: Relative humidity
IOD: Integrated optical density
AOD: Average optical density
TGF-β: Transforming growth factor beta
ANOVA: A one-way analysis of variance
ATT: α-1 antitrypsin
